# Association between cognitive function and body composition in older adults: data from NHANES (1999–2002)

**DOI:** 10.3389/fnagi.2024.1372583

**Published:** 2024-03-20

**Authors:** Lianghua Chen, Liling Zou, Jingwen Chen, Yixiao Wang, Dandan Liu, Lianjun Yin, Junqi Chen, Haihong Li

**Affiliations:** ^1^Department of Rehabilitation Medicine, The Third Affiliated Hospital of Southern Medical University, Guangzhou, Guangdong Province, China; ^2^Department of Rehabilitation Medicine, The Sixth People’s Hospital of Nanhai District, Foshan, Guangdong Province, China

**Keywords:** cognitive function, body composition, total bone mineral density, National Health and Nutrition Examination Survey, older adults

## Abstract

**Aim:**

To investigate the association between cognitive function and body composition in older adults.

**Methods:**

We collected data on 2080 older adults (>60 years of age) from the National Health and Nutrition Examination Survey (NHANES) for the years 1999–2000 and 2001–2002. Candidate variables included: demographic data (sex, age, race, education level, marital status, poverty-to-income ratio), alcohol consumption, cardiovascular disease, diabetes, osteoporosis, total bone mineral density, and total fat mass. A logistic regression model was established to analyze the association between cognitive function and body composition in older adults. In addition, stratified logics regression analysis was performed by sex and age.

**Results:**

Bone mineral density significantly affects cognitive function in older adults (*p*<0.01). When examining the data according to sex, this correlation is present for women (*p* < 0.01). For men, though, it is not significant (*p* = 0.081). Stratified by age, total bone mineral density was significantly correlated with cognitive function in 60–70 and 70–80 years old people, but not in older adults older than 80 years(for 60–70 years old, *p* = 0.019; for 70–80 years old, *p* = 0.022). There was no significant correlation between total bone mineral density and cognitive function (*p* = 0.575).

**Conclusion:**

The decrease of total bone mineral density was significantly correlated with cognitive decline in the older adults, especially among women and older people in the 60 to 80 age group. There was no connection between total fat mass, total percent fat, total lean mass, appendicular lean mass, appendicular lean mass /BMI and cognitive function in the older adults.

## 1 Introduction

Cognitive decline often occurs with aging and is caused by many factors acting simultaneously. It is an important problem among older adults and has a serious impact on their lives. Physical conditions in older adults, such as muscle and bone loss, are receiving more attention. These issues are getting more significant (Haasum et al., 2012; Colón et al., 2018; Dionyssiotis, 2019). Understanding these health issues requires an understanding of body composition. It assists in evaluating both physical and nutritional health. The assessment generally concentrates on lean tissue, fat, and bone. There is a close relationship between changes in cognitive function and age, and as we age, we can usually observe a concomitant change in body composition that leads to a decrease in physical function; whether this change is associated with reduced or impaired cognitive function is not widely studied. Research indicates a connection between variables including body fat percentage and bone mineral density and cognitive deterioration. The evidence is still weak, though. To validate these links, further thorough investigation is required. Moreover, the existing studies often exploring the association between one type of body composition and cognitive function individually, without exploring the association between different body compositions jointly.

To fill the research gap and build on existing studies, this study will examine changes in cognitive function along three dimensions: bone, adiposity, and fat-free soft tissue, while controlling for other risk factors (sex, age, race, education level, marital status, poverty-to-income ratio, alcohol consumption status, history of cardiovascular disease, history of diabetes, history of osteoporosis, etc.) that may have an impact on cognitive function association with body composition. Exploring the association between cognitive function and body composition in older adults from multiple dimensions cannot only explore the relationship between body composition and cognitive function, but also better observe whether different body compositions are independently associated with cognitive function in older adults by controlling for the effects of different body compositions as well as other factors on cognitive function. The study was conducted to fill the gap in the current study and to add evidence to the study of factors related to cognitive decline in the older adults. Dementia in older adults can be prevented by managing and addressing issues that affect cognitive performance early on. With this strategy, the prevalence of dementia in clinical settings is to be reduced.

## 2 Materials and methods

### 2.1 Data sources

We gathered all study data from the Centers for Disease Control and the National Center for Health Statistics (NCHS). The National Health and Nutrition Examination Survey (NHANES) was the primary source of the data. Two rounds of this survey were carried out: in 1999–2000 and in 2001–2002. The objective of this program is to evaluate the nutritional and overall health of adults and children in the United States. The NHANES program began in the early 1960’s to collect information on the health and nutrition of the U.S. household population. The program surveys a nationally representative sample of approximately 5,000 people each year, came from counties throughout the United States. The program consists of an interview component and a physical examination component. The interview component includes questions related to demographics, socioeconomics, diet, and health. The physical examination component includes physiological measurements, laboratory tests, and other components. The results will be used to determine the prevalence of major diseases and risk factors of diseases, as well as to assess the nutritional status and health of different populations. The Centers for Disease Control and NCHS’s Ethics Board approved the surveys. All participants gave their written consent.

### 2.2 Study participants

A total of 21,004 participants were included in the National Health and Nutrition Examination Survey (NHANES) 1999–2000 and 2001–2002 survey cohorts. From which a total of 3,706 participants aged 60 years old were identified in this study. Participants were excluded if they lacked data on cognitive function tests (*n* = 731), dual-energy X-ray absorptiometry (DXA) examinations (*n* = 349) were excluded. Of the resulting 2,626 participants, 546 participants were ultimately excluded for lack of data on one or more of the following variables: race, education, marital status, poverty-income ratio, body mass index, alcohol consumption, smoking, lipid profile, history of cardiovascular disease, history of osteoporosis, history of fracture, and history of diabetes mellitus. Hence, the ultimate study cohort for this research comprised 2080 elderly individuals who satisfied all inclusion criteria and possessed comprehensive analytical data for statistical examination.

### 2.3 Cognitive functioning test

**Figure 1:**
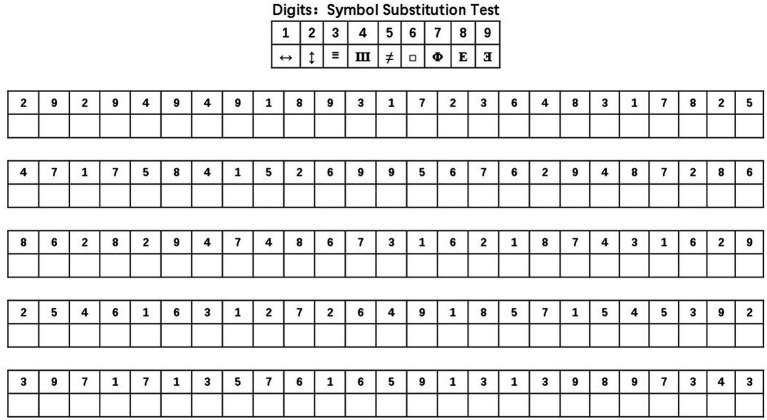
DSST test symbol coding table. DSST, digit symbol substitution test, that is based on a digital-symbol coding table, where each digit from 1 to 9 corresponds to a symbol. The subjects matched symbols to numbers as many times as they could in a coding table for 90 s, and the higher the score, the better the cognitive function.

The Digit Symbol Substitution Test (DSST), a cognitive function assessment, is administered to NHANES study participants 60 years of age and older. The DSST, which is a component of the Wechsler Adult Intelligence Scale, assesses executive function, memory, attention, and brain speed (Tsatali et al., 2021). The DSST test (Figure 1) is completed primarily by having the subject match numbers and symbols on a test sheet. The paper on which the DSST is administered has nine symbols arranged by numbers 1–9 at the top and 100 spaces with different numbered digits at the bottom (Wechsler, 1997; Jaeger, 2018). At the beginning of the test, the participant is asked to copy the symbols under the top number into the corresponding numbered spaces below. Within the time allowed (usually 90 to 120 s), the number of correct symbols completed by the subject constitutes a score that ranges from 0 to 133 after the test is completed, and this score is the sum of the scores of correctly matching numerical symbols. Currently, there is no gold standard for the DSST test to identify low cognitive performance. Therefore, the 25th percentile of the total score was set as the low cognitive function cutoff, which is consistent with the approach used in the published literature (Rosano et al., 2016; Chen et al., 2017). In the present study a DSST test cut-off score of 30 was used in the study sample, and we used this as a basis for dividing the sample into two groups: the low cognitive functioning group and the normal cognitive functioning group.

### 2.4 DXA for body composition

We measured the study population’s body composition using dual-energy X-ray absorptiometry (DXA). DXA is popular because it’s fast, easy to use, and has low radiation. DXA examinations by NHANES provided overall information about body composition (bone and soft tissue) as well as nationally representative data on age, sex, and race to examine the association between body composition and other health conditions and risk factors. During the DXA exam, all participants put on exam-specific clothing and remove jewelry and other personal items that could interfere with the DXA test. DXA test results were reviewed and analyzed by the UCSF Department of Radiology Bone Density Group using industry standard techniques. All DXA results that may affect the accuracy of DXA results such as prosthetic devices, implants or other extraneous objects will be set to missing in the data set for the corresponding subject. DXA scans provide skeletal and soft tissue measurements of the whole body, both arms and legs, trunk and head. Skeletal measurements of the pelvis, right and left ribs, thoracic and lumbar spine are also obtained. Measurements for the whole body and body regions included total mass (g), bone mineral content (BMC) (g), bone area (cm^2^), bone mineral density (BMD) (g/cm^2^), fat mass (g), lean body mass excluding bone mineral content (g), lean body mass including bone mineral content (g), and fat percentage (%).

The index chosen for this study regarding bone was mainly whole-body bone mineral density, which provides a comprehensive view of the entire skeleton and is mainly determined by cortical bone (Iori et al., 2019). The cortical bone is associated with fractures in aging. For older adult population, decreased cortical bone density is more specific than trabecular bone density and may play an important role in bone fragility in the older adults (Almeida et al., 2017). Whole body fat percentage is considered to be an important indicator of obesity, therefore, in this study, whole body fat percentage was chosen to explore the association between body composition fat mass and cognitive function. In the National Institutes of Health Muscular Dystrophy Program (Liao et al., 2018), lean body mass of the extremities adjusted with body mass index – lean body mass of the extremities/body mass index – was used as a Sarcopenic. In addition, unadjusted limb lean body mass and whole-body lean body mass were also included to investigate the association between fat free soft tissue and cognitive function.

In summary, whole-body bone mineral density, whole-body lean body mass, whole-body limb weight, whole-body fat mass, and whole-body fat percentage were selected as the primary parameters. Furthermore, the lean muscle mass/body mass index of the limbs was calculated based on the relevant measurements and included in this study. In addition, the mass unit (g) was converted to (kg) in this study (except for bone mineral density).

### 2.5 Covariates

In addition to cognitive function and body composition-related indicators, factors that may affect cognitive function, such as sex, age, race, education level, marital status, poverty-to-income ratio, alcohol consumption status, history of cardiovascular disease, history of diabetes mellitus, and history of osteoporosis were selected as covariates. The above data were obtained from the NHANES database.

### 2.6 Statistical analysis

All data were analyzed statistically using SPSS26.0 software. In this study, the types of data included continuous and categorical variables, and we used “mean ± standard deviation (x̅ ± SD)” to describe continuous variables that conformed to a normal distribution, and “median (interquartile range) (M (IQR)).” If the variable was a categorical variable, it was described using the number of cases *n* (%). We conducted univariate analyses of the relationship between all variables and cognitive functioning. In the univariate analysis, the independent samples *t*-test was used to compare the mean between the low and normal cognitive function groups when the variables conformed to a normal distribution, and the Mann–Whitney U test was used when the variables did not conform to a normal distribution. In addition, the chi-square test or Fisher’s exact probability method was used to compare the percentages of categorical variables. Candidate variables with *p* < 0.1 in the univariate analysis were subsequently included in a multifactorial logistic regression model to further analyze the association between cognitive function and physical components in older adults. Candidate variables included were: demographics (sex, age, race, education level, marital status, poverty-to-income ratio), alcohol consumption status, history of cardiovascular disease, history of diabetes, history of osteoporosis, total body bone mineral density, and total body fat mass. In our final logistic regression analysis exploring the association between cognitive function and body composition in older adults, Model 1 was not adjusted for any confounders, Model 2 was adjusted for age and sex, and Model 3 was adjusted for race, education level, marital status, poverty-income ratio, alcohol use status, history of diabetes, history of cardiovascular disease, and history of osteoporosis. We then conducted stratified logistic regression analyses by sex and age to further explore the association between cognitive function and physical components in older adults across dimensions. All variables in the logistic regression models were tested for covariance and did not have cointegration. A two-sided *p* < 0.05 was considered statistically significant.

## 3 Results

### 3.1 Characteristics of study population

The study sample was split into two groups based on the lowest percentile of DSST test scores (30 in this study), with a total of 2080 persons included in the study populations. All participants were above 60 years old. The group with low cognitive functioning (*n* = 504) and the group with normal cognitive functioning (*n* = 1,576) were identified by DSST scores less than 30. These results showed that the population characteristics of older, non-Hispanic white, less educated, poorer, never married, and almost never drink alcohol were more likely to be low cognitive functioning, and the prevalence of diabetes was higher in the low cognitive functioning group than in the normal cognitive functioning group. There were no statistically significant differences in sex distribution, body mass index, smoking status, history of cardiovascular disease, history of hyperlipidemia, and history of fracture between the two groups (Table 1).

**Table 1:** Characteristics of the study population.

Crowd characteristics	Normal cognitive function group (*n* = 1,576)	Low cognitive function group (*n* = 504)	*X* ^2^	*p*
Sex			2.952	0.086
Male, *n* (%)	775 (49.2%)	270 (53.6%)		
Female, *n* (%)	801 (50.8%)	234 (46.4%)		
Age			31.681	<0.01^*^
60–70 years old, *n* (%)	791 (50.2%)	196 (38.9%)		
70–80 years old, *n* (%)	525 (33.3%)	173 (34.3%)		
≥80 years old, *n* (%)	260 (16.5%)	135 (26.8%)		
Race			224.522	<0.01^*^
Mexican American, *n* (%)	1,091 (65.2%)	165 (32.7%)		
Other Hispanic, *n* (%)	190 (12.1%)	121 (24.0%)		
Non-Hispanic White, *n* (%)	220 (14.0%)	178 (35.3%)		
Non-Hispanic Black, *n* (%)	30 (1.9%)	8 (1.6%)		
Other race, *n* (%)	45 (2.9%)	32 (6.3%)		
Education level			410.546	<0.01^*^
Less than high school, *n* (%)	420 (26.6%)	385 (76.4%)		
High school graduate, *n* (%)	437 (27.7%)	74 (14.7%)		
High school or higher, *n* (%)	719 (45.6%)	45 (8.9%)		
Marital status			26.778	<0.01^*^
Married/live with partner, *n* (%)	1,044 (66.2%)	272 (54.0%)		
Divorced/separated/widowed, *n* (%)	502 (31.9%)	213 (42.3%)		
Never married, *n* (%)	30 (1.9%)	19 (3.8%)		
Poverty to income ratio			173.296	<0.01^*^
≤0.99, *n* (%)	142 (9.0%)	166 (32.9%)		
≥1, *n* (%)	1,434 (91.0%)	338 (67.3%)		
Body mass index			0.157	0.924
<25 kg/m^2^, *n* (%)	438 (27.8%)	139 (27.6%)		
25–30 kg/m^2^, *n* (%)	646 (41.0%)	203 (40.3%)		
≥30 kg/m^2^, *n* (%)	492 (31.2%)	162 (32.1%)		
Smoking			0.945	0.623
Hardly smoked, *n* (%)	727 (46.1%)	242 (48.0%)		
Ever smoked, *n* (%)	664 (42.1%)	200 (39.7%)		
Current smoker, *n* (%)	185 (11.7%)	62 (12.3%)		
Drinking alcohol			19.192	<0.01^*^
Hardly drink alcohol, *n* (%)	255 (16.2%)	123 (24.4%)		
Rarely drink alcohol, *n* (%)	318 (20.2%)	105 (20.8%)		
Regular drinker, *n* (%)	1,003 (63.6%)	276 (54.8%)		
Cardiovascular disease, *n* (%)	354 (22.5%)	132 (26.2%)	2.965	0.085
Hyperlipidemia, *n* (%)	1,228 (77.9%)	375 (74.4%)	2.668	0.102
Diabetes mellitus, *n* (%)	223 (14.1%)	124 (24.6%)	30.022	<0.01^*^
Osteoporosis, *n* (%)	192 (12.2%)	32 (6.3%)	13.523	<0.01^*^
History of fracture, *n* (%)	203 (12.9%)	71 (14.1%)	0.486	0.486

Normal cognitive function group: DSST scores above 30. Low cognitive function group: DSST scores below 30. * *p* < 0.05.

### 3.2 Body composition in different cognitive function groups

As seen in Table 2, the median whole-body bone mineral density of the low cognitive function group was 1.035 g/cm^3^, the median whole-body fat mass was 26.41 kg, the median whole-body fat percentage was 35.6%, the median whole-body lean body mass was 46.08 kg, the median limb lean body mass was 19.53 kg, the median limb lean body mass/body mass index was 0.700, the median normal cognitive, the median total body bone mineral density in the functional group was 1.063 g/cm^3^, the median total body fat was 27.36 kg, the median total body fat percentage was 36.2%, the median total body lean body mass was 46.84 kg, the median limb lean body mass was 19.81 kg, and the median limb lean body mass/body mass index was 0.705. In the Mann–Whitney U test, whole-body bone mineral density (Z = −3.917, *p* < 0.01) and whole-body fat mass (Z = −32.659, *p* < 0.01) were significantly reduced in older adults in the low cognitive function group compared with the normal cognitive function group, and high whole-body bone mineral density as well as high whole-body fat mass may have a protective effect on cognitive function, while the Percentage of whole-body fat mass (Z = −1.316, *p* = 0.188), whole-body lean body mass (Z = −1.278, *p* = 0.201), limb lean body mass (Z = −1.236, *p* = 0.217), and limb lean body mass/body mass index (Z = 1.467, *p* = 0.142) were not significantly different in the low cognitive function group compared with the normal cognitive function group.

**Table 2:** Body composition in different cognitive function groups.

Variables	Normal cognitive function group (*n* = 1,576)	Low cognitive function group (*n* = 504)	*X* ^2^	*p*
Whole-body bone mineral density, M(IQR), g/cm^3^	1.063 (0.971–1.061)	1.035 (0.938–1.137)	−3.917	<0.01^*^
Whole-body fat mass, M(IQR), kg	27.36 (22.22–33.87)	26.41 (20.97–33.04)	−2.659	<0.01^*^
Whole body fat mass percentage, M(IQR), %	36.2 (30.3–42.7)	35.6 (30.0–42.2)	−1.316	0.188
Whole-body lean body mass, M(IQR), kg	46.84 (38.51–55.65)	46.08 (38.95–53.53)	−1.278	0.201
Extremity lean body mass, M(IQR), kg	19.81 (15.77–24.18)	19.53 (15.84–23.11)	−1.236	0.217
Extremity lean body mass/body mass index, M(IQR)	0.705 (0.572–0.866)	0.700 (0.559–0.842)	−1.467	0.142

Normal cognitive function group: DSST scores above 30. Low cognitive function group: DSST scores below 30. M (IQR), Median (Interquartile range). * *p* < 0.05.

### 3.3 Logistic regression model of the association between cognitive function and body composition

We included variables in the logistic regression if they had a *p*-value of less than 0.1 in the initial analysis. These variables were sex, age, race, education, marital status, income, alcohol use, and histories of cardiovascular disease, diabetes, and osteoporosis, along with whole-body bone density and fat mass. Using logistic regression models, we examined the relationship between bone density, body fat, and cognitive performance. Initially, we utilized unmodified models. Next, we compared those findings with models that just took age and sex into account. As seen in Table 3, the OR (95% CI) for whole-body bone mineral density was 0.193 (0.092–0.405) in model 1, 0.085 (0.033–0.218) in model 2, and 0.154 (0.046–0.509) in model 3, with *p* values were < 0.01, which shows that after adjusting for all included covariates, the association between whole-body BMD and cognitive function remained significant and negatively correlated, with lower whole-body BMD being more likely to be associated with lower cognitive function. The OR (95% CI) for whole-body adiposity was 1.000 (1.000–1.000) in either model 1, model 2, and model 3 and was not associated with cognitive function.

**Table 3:** Logistic regression model of the association between cognitive function and body composition.

	Model 1			Model 2			Model 3		
	OR	95%CI	*p*	OR	95%CI	*p*	OR	95%CI	*p*
Whole body fat mass	1.000	0.999–1.000	0.013^*^	1.000	0.999–1.000	0.291	1.000	0.999–1.000	0.646
Whole-body bone mineral density	0.193	0.092–0.405	<0.01^*^	0.085	0.033–0.218	<0.01^*^	0.154	0.046–0.509	<0.01^*^

Model 1 = a logistic regression model without adjustment for covariates.

Model 2 = a logisticregression model with adjustment for age and sex.

Model 3 = a logistic regression model with adjustment for sex, age, race, education level, marital status, poverty-to-income ratio, alcohol consumption status, history of cardiovascular disease, history of diabetes, and history of osteoporosis.

OR, odds ratio; CI, confidence interval. * *p* < 0.05.

### 3.4 The association between cognitive function and body composition in older adults in a sex-stratified logistic regression model

As seen in Table 4, the OR (95% CI) for whole-body adiposity was 1.000 (1.000–1.000) in both the male and female populations and was not considered to be associated with cognitive function. The OR (95% CI) for whole-body bone mineral density was 0.238 (0.047–1.196) in males and 0.067 (0.010–0.424) in females, which tells that in males, whole-body bone mineral density was not significantly correlated with cognitive function and in female’s whole-body bone mineral density was significantly correlated with cognitive function.

**Table 4:** Logistic regression model of the association between cognitive function and body composition based on sex.

	Male			Female		
	OR	95%CI	*p*	OR	95%CI	*p*
Whole body fat mass	1.000	1.000–1.000	0.824	1.000	1.000–1.000	0.493
Whole-body bone mineral density	0.238	0.047–1.196	0.081	0.067	0.010–0.424	<0.01^*^

OR, odds ratio; CI, confidence interval. * *p* < 0.05.

### 3.5 The association between cognitive function and body composition in older adults in an age-stratified logistic regression model

An age-stratified logistic regression model was created for this study’s analysis in order to investigate the relationship between cognitive performance and body composition in various age groups, as seen in Table 5. The OR (95% CI) for whole-body adiposity was 1.000 (1.000–1.000) at all ages, which was considered to be unrelated to any change in cognitive function. The OR (95% CI) for whole-body bone mineral density was 0.099 (0.014–0.687) in those aged 60–70 years, 0.075 (0.008–0.692) in those aged 70–80 years, and 0.523 (95% CI) in those aged >80 years (0.054–5.034). It may be seen that whole-body BMD was strongly correlated with cognitive function in those between the ages of 60 and 80, but not significantly correlated with cognitive performance in those above 80.

**Table 5:** Logistic regression model of the association between cognitive function and body composition based on age.

	60–70 years			70–80 years			≥80 years		
Variables	OR	95%CI	*p*	OR	95%CI	*p*	OR	95%CI	*p*
Whole body fat mass	1.000	1.000–1.000	0.048	1.000	1.000–1.000	0.266	1.000	1.000–1.000	0.406
Whole-body bone mineral density	0.099	0.014–0.687	0.019^*^	0.075	0.008–0.692	0.022^*^	0.523	0.054–5.034	0.575

OR, odds ratio; CI, confidence interval. * *p* < 0.05.

## 4 Discussion

### 4.1 Association between cognitive function and bone

Our research demonstrates the association between reduced cognitive performance and lower whole-body bone mineral density (BMD). This is especially applicable for women and people in their 60s to 80s. In our investigation, we took other risk variables into account. This is consistent with previous findings. The association between BMD and cognitive function was not attenuated when we controlled for sex, age, race, education level, marital status, poverty to income ratio, alcohol use, history of cardiovascular disease, history of diabetes, history of osteoporosis, and total body fat mass. This evidence suggests that bone loss in older adults is associated with cognitive function, but it is unclear whether this change is caused by altered cognitive function, or whether bone loss in older adults increases the risk of reduced cognitive function, or whether there may be a common biological mechanism for bone loss and cognitive function in older adults. Evidence from earlier studies shows that estrogen is linked to changes in cognitive function as well as reduced bone mineral density (Daniel et al., 2015), so the association between reduced bone mineral density and altered cognitive function may be caused by estrogen. Estrogen may play an important role in preventing osteoporosis and cognitive function. It delays neuronal apoptosis and atherosclerosis, inhibits the formation of amyloid b protein, prevents hyperphosphorylation of tau protein, reduces oxidative stress response in the brain, and improves brain cell metabolism (Jett et al., 2022; Zhang et al., 2022). The findings of Loskutova et al. (2010) suggested that BMD is reduced in both men and women in the early clinical stages of Alzheimer’s disease, and low BMD may be useful for identifying participants with Alzheimer’s disease or cognitive decline. Although we did not include estrogen levels in our study, our findings also suggest that the association between reduced bone mineral density and cognitive function is more significant in women compared to men, as well as recent clinical trial evidence suggesting an increased risk of dementia in estrogen users (Stefanidou et al., 2021), indicating that estrogen levels may be responsible for the association of bone loss with cognitive function.

In addition, several epidemiological studies suggest that dietary factors may contribute a role in reduced cognitive function in older adults (Beauchet et al., 2019; McGrattan et al., 2019; van den Brink et al., 2019). Although the role of dietary protein and fat in bone health remains a controversial issue, animal and human studies suggest that an adequate supply of energy and protein is essential to maintain bone health in older adults (Deane et al., 2020; Liu et al., 2023). Reduced food intake and altered dietary patterns may occur in older adults with low cognitive function and may explain the reduced BMD in the low cognitive function group. In addition to dietary deficiencies in micronutrients and nutrients, disturbances in micronutrient metabolism and exposure to high concentrations of certain minerals have been associated with low bone mass and Alzheimer’s disease (Ali et al., 2019; Lei et al., 2021; Martins et al., 2021). Although diet was not assessed in this study, the similarity of body mass index and body fat percentage between the low and normal cognitive function groups in the study sample and the absence of statistical differences in the univariate analysis suggest a similar nutritional status between the low and normal cognitive function groups; therefore, we infer that a strong association between bone loss and cognitive function caused by dietary patterns is less likely.

Several studies have shown that the APOE4 allele increases the risk of progression of cognitive impairment to Alzheimer’s-type dementia more than fourfold (*p* < 0.001), and it is known that the APOE4 allele is associated with both bone loss and cognitive impairment (Macdonald et al., 2008; Zachara et al., 2022), so we can infer that the association between reduced bone mineral density and association between reduced BMD and reduced cognitive function may result from the action of the APOE4 allele.

Another possible explanation for the present results is that neurodegenerative brain changes associated with cognitive decline may directly affect the central control of bone remodeling. Atrophy of the limbic system, including the hypothalamus, is prominent in Alzheimer’s disease (Weiner et al., 2017), and the hypothalamus is a central regulator of many homeostatic metabolic processes playing a major role in the regulation of bone mass (Zaidi, 2007; Idelevich and Baron, 2018). This may be a factor contributing to the association between bone loss associated with cognitive decline.

### 4.2 Association between cognitive function and body fat

After controlling risk factors that may have an impact on cognitive function (sex, age, race, education level, marital status, poverty-to-income ratio, alcohol consumption status, history of cardiovascular disease, history of diabetes, history of osteoporosis, etc.), our findings did not reveal a significant association between cognitive impairment and body fat content and percentage of body fat in older adults. Nevertheless, this is inconsistent with the results of some studies. Large-scale epidemiological studies have confirmed that cardiovascular risk factors are associated with cognitive impairment (Hagi et al., 2021). Obesity is one of the cardiovascular risk factors, may affect cognitive function through cardiovascular health but may also have independent effects on cognitive function, such as the induction of pro-inflammatory factors leading to systemic hypo-inflammation, and systemic hypo-inflammation is thought to be a possible trigger for the development of Alzheimer’s disease (Spyridaki et al., 2014). Higher levels of obesity are associated with low-grade systemic inflammation, dyslipidemia, hypertension, and diabetes, which in turn are associated with small vessel disease in the form of high white matter signaling. Together they are associated with subcortical and cortical alterations of obesity and cognitive deficits (Dadar et al., 2021). Studies have shown that obesity leads to accelerated brain aging and is a risk factor for dementia. This has clinical implications for obesity management and dementia prevention. However, in this study neither body mass index, one of the indicators of obesity, nor total body fat mass and total body fat ratio were associated with cognitive decline, as well as history of hyperlipidemia and cardiovascular disease were not associated with cognitive function. In the univariate analysis, it was suggested that there was no statistical difference in body mass index and whole-body fat percentage between the low and normal cognitive function groups. In a cross-sectional study involving 9,189 adults aged 30–75 years, in which cognitive function was also assessed by DSST test scores, the results of this study showed that body fat percentage was significantly associated with cognitive function (Anand et al., 2022) and that cognitive function decreased with increasing body fat percentage. This contradicts the findings of our investigation, and because of the small sample size, we are unable to rule out the possibility that the sample contributed to the variation in outcomes. Our study’s age range was also different, and it is possible that this had an impact on the variations in the findings. In addition, the above study was conducted mainly in the Canadian population, and it is also possible that the differences in the study results were due to differences in ethnic groups. Whether body fat percentage, for example, is associated with cognitive function, our findings cannot be certain and we cannot exclude that there is an error due to sample selection. However, in our findings, no association was found between body mass index, total body fat mass, body fat percentage, history of hyperlipidemia, history of cardiovascular disease, and cognitive function in older adults.

### 4.3 Association between cognitive function and body fat-free soft tissue

According to our research, there is no correlation between an older adult’s body mass index, limb lean body mass, or whole-body lean body mass with cognitive performance. The available research evidence suggests that sarcopenia is closely associated with reduced cognitive function, and that the diagnostic criteria for sarcopenia are muscle mass as well as muscle function (Du et al., 2023), although individual studies have suggested that reduced muscle mass may be associated with reduced cognitive function, and an observational prospective initial cohort study showed that low cognitive function in older adults inpatients was associated with low grip strength and low lean body mass (van Dam et al., 2018). Most studies have concluded that muscle function is strongly associated with cognitive function. These findings are consistent with evidence linking gait performance, grip strength, and cognitive impairment (McGrath et al., 2020; Pang et al., 2021). Due to limitations in data sources, our study did not explore the relationship between muscle function indicators such as gait performance and grip strength and cognitive function, but mainly explored the relationship between muscle mass and cognitive function, and the apparent lack of independent associations between low muscle mass and cognitive function in our findings is consistent with the majority of studies. The mechanisms underlying the correlation between muscle function and cognitive function remained unclear, but the existence of common pathological factors such as chronic inflammation, oxidative stress, hormonal alterations and insulin resistance have been hypothesized to explain the relationship between poor muscle function and cognitive decline, and since gait performance and grip strength changes all involve multiple brain regions (Pang et al., 2021). There is a hypothesis that suggests comparable neural pathways may be responsible for the association observed between cognitive and muscular function.

## 5 Conclusion

This study used information from the NHANES database to perform a cross-sectional study to look at the link between cognitive function and body composition in older adults aged >60 years (*n* = 2080). The findings indicate that even after adjusting for various potential confounding factors such as sex, age, race, education level, marital status, poverty-to-income ratio, alcohol consumption status, history of cardiovascular disease, history of diabetes, and osteoporosis, a reduced whole-body bone mineral density in older adults was still significantly associated with cognitive decline, especially in women and in those aged 60–80 years.

## Data availability statement

The datasets presented in this study can be found in online repositories. The names of the repository/repositories and accession number(s) can be found at: NHANES (1999–2002).

## Ethics statement

The studies involving humans were approved by National Health and Nutrition Examination Survey (NHANES). The studies were conducted in accordance with the local legislation and institutional requirements. Written informed consent for participation in this study was provided by the participants’ legal guardians/next of kin. Written informed consent was obtained from the individual(s) for the publication of any potentially identifiable images or data included in this article.

## Author contributions

LC: Writing – review & editing, Writing – original draft. LZ: Writing – review & editing, Writing – original draft. JiC: Writing – original draft, Software, Formal analysis, Data curation. YW: Writing – original draft, Software, Formal analysis. DL: Writing – original draft, Data curation. LY: Writing – original draft, Data curation. JuC: Writing – review & editing. HL: Writing – review & editing.
